# A qualitative exploration of health care workers’ approaches to relational harm reduction in HIV primary care settings

**DOI:** 10.1186/s12954-024-01021-x

**Published:** 2024-05-17

**Authors:** Emma Sophia Kay, Stephanie L. Creasy, Jessica Townsend, Mary Hawk

**Affiliations:** 1 School of Nursing, University of Alabama at Birmingham, 1701 University Blvd., Birmingham, Alabama, AL 35294 USA; 2 Department of Behavioral and Community Health Sciences, School of Public Health, University of Pittsburgh, Pittsburgh, PA USA

## Abstract

**Background:**

Structural harm reduction is an approach to care for people who use drugs (PWUD) that incorporates services and resources (e.g., naloxone, sterile syringes). As conceptualized in our previous research, harm reduction is also “relational,” encompassing a patient-provider relationship that is non-judgmental and respectful of patients’ autonomy. Little is known about health care workers’ (HCW) knowledge or attitudes towards harm reduction beyond structural strategies, whose availability and legality vary across geographical settings. To operationalize how relational harm reduction is both characterized and employed in HIV care settings, where nearly half of patients have a diagnosed substance use disorder, we qualitatively explored HCWs’ knowledge of and use of harm reduction via individual in-depth interviews.

**Methods:**

Our study sample included three HIV clinics, one in Birmingham, Alabama (AL) and two in Pittsburgh, Pennsylvania (PA). We conducted individual interviews with *n* = 23 health care workers via Zoom, using a semi-structured interview guide to probe for questions around health care workers’ attitudes towards and experiences with providing care to PWH who use drugs and their knowledge of and attitudes towards relational and structural harm reduction. Data was analyzed in Dedoose using thematic analysis.

**Results:**

Qualitative analyses revealed two primary themes, *Continuum of Relational Harm Reduction in Practice and Limited Harm Reduction Training*. Nearly all HCWs (*n* = 19, 83%) described a patient interaction or expressed a sentiment that corresponded with the principles of relational harm reduction. Yet, over half of participants (*n* = 14, 61%) used language to describe PWH who use drugs that was stigmatizing or described an interaction that was antithetical to the principles of relational harm reduction. Five HCWs, all from Birmingham, were unaware of the term ‘harm reduction.’ Few HCWs had any harm reduction training, with most learning about harm reduction from webinars/conferences or on the job.

**Conclusion:**

Our findings suggest that relational harm reduction in HIV care settings is practiced along a continuum, and that a range of behaviors exist even within individual HCWs (e.g., used stigmatizing terms such as “addict” but also described patient interactions that reflected patients’ autonomy). Given that harm reduction is typically described as a structural approach, a broader definition of harm reduction that is not dependent on policy-dependent resources is needed.

**Supplementary Information:**

The online version contains supplementary material available at 10.1186/s12954-024-01021-x.

## Introduction

Harm reduction is an approach to care developed by people who use drugs (PWUD) for PWUD that incorporates not only services and resources (e.g., naloxone, sterile syringes, fentanyl test strips—structural harm reduction), but also patient-provider relationships that are non-judgmental and respectful of patients’ autonomy, defined as relational harm reduction [1]. Harm reduction is aimed at minimizing harm associated with drug use, rather than requiring abstinence. In a previous study, we outlined a set of six harm reduction principles in medical settings, which can be used to guide health care workers’ (HCW) interactions with patients [1] (See Table 1 for a list of the relational harm reduction principles and their definitions.) The purpose of this work was to operationalize ways that HCW can apply long-standing harm reduction principles in the context of their work. As these principles are the foundation of health care workers’ communication with PWUD, we define the operationalization of these principles as “relational” harm reduction, while we refer to drug overdose prevention strategies and other tangible services as “structural” harm reduction. While the two forms of harm reduction should ideally be paired in practice, we delineate them here to underscore that the ways in which care is delivered is as important as the specific services provided. Moreover, there is variability of structural harm reduction services across different political contexts (e.g., seven states, including Alabama, one of our study locations, have failed to legalize sterile syringe programs to date [2]), while relational harm reduction can be practiced in any political context.

**Table 1:** Harm reduction principles and definitions

Humanism	• Providers value, care for, respect, and dignify patients as individuals.
	• It is important to recognize that people do things for a reason; harmful health behaviors provide some benefit to the individual and those benefits must be assessed and acknowledged to understand the balance between harms and benefits.
	• Understanding why patients make decisions is empowering for providers.
Pragmatism	• None of us will ever achieve perfect health behaviors.
	• Health behaviors and the ability to change them are influenced by social and community norms; behaviors do not occur within a vacuum.
Individualism	• Every person presents with their own needs and strengths.
	• People present with spectrums of harm and receptivity and therefore require a spectrum of intervention options.
Autonomy	• Though providers offer suggestions and education regarding patients’ medications and treatment options, individuals ultimately make their own choices about medications, treatment, and health behaviors to the best of their abilities, beliefs, and priorities.
Incrementalism	• Any positive change is a step toward improved health, and positive change can take years.
	• It is important to understand and plan for backward movements.
Accountability without termination	• Patients are responsible for their choices and health behaviors.
	• Patients are not “fired” for not achieving goals.
	• Individuals have the right to make harmful health decisions, and providers can still help them to understand that the consequences are their own.

A recent editorial by the director of the National Institute on Drug Abuse (NIDA) urges HCWs to provide compassionate, non-stigmatizing care to PWUD, noting the alternative may exacerbate drug use [3]. Continued conflation of drug use as “abuse,” which implies that any drug use is wrong, pervades social messaging [4]. Indeed, research shows that HCWs are not immune from this social messaging, with some providers regarding PWUD as “criminal” [5]. To avoid experiences of stigma and discrimination when receiving health care services, PWUD may seek to avoid stigma by concealing their drug use from HCWs, minimizing symptoms of pain, and even delaying care altogether [6, 7]. PWUD may even avoid calling emergency medical services for fear of arrest [8]. These negative experiences in healthcare settings decrease trust in the medical system, raising risk of adverse health outcomes such as death from injection-related infections [7], relying on non-prescription medication to alleviate pain [9], and leaving the hospital against medical advice [10]. However, patients who feel respected by and trust their HCWs are more likely to experience positive health outcomes [22–27]. For PWUD, greater trust in their provider is associated with positive expectations for their interactions with their providers and is mediated by perceived provider support for harm reduction [11]. PWUD also cite the harm reduction principles of humanism, pragmatism, autonomy, individualism, incrementalism, and autonomy without termination [1], as well as ongoing support, reliability, and provider expertise in treating substance use disorder [12], as cornerstones of strong patient-provider relationships.

To better serve the needs of PWUD, scholars and providers have recommended integrating harm reduction into primary care and other settings that do not explicitly serve PWUD and have recognized the importance of the patient-provider relationship as a form of harm reduction [13]. Indeed, harm reduction has been recognized as one of the key components of the US Department of Health and Human Services’ Overdose Prevention Strategy [14], and the Health and Medicine panel of the National Academy of Sciences, Engineering and Medicine has recommended incorporating harm reduction strategies into infectious disease and opioid use disorder care [15]. Since the elimination of the X-Waiver in 2023, any provider can prescribe buprenorphine without having to register with the Drug Enforcement Administration [16], a requirement that was previously noted as a significant barrier to prescribing medication for opioid use disorder (MOUD) [17]. Yet, despite the importance of integrating harm reduction principles into health care settings, little is known about what this looks like in practice. Extant literature is focused on HCWs’ knowledge of structural harm reduction services. For example, a previous scoping review focusing on harm reduction for people who use opioids identified 25 studies that examined physicians’ knowledge and perceptions of harm reduction for people who use opioids [18]. Knowledge gaps include those related to prescribing medication for opioid use disorder and using naloxone, and uncertainly about their legality. Physicians’ perceptions of harm reduction highlighted the prevalence of stigma and concerns about medication diversion [18]. Finally, the scoping review revealed system and institutional barriers to the provision of high-quality care for PWUD, such as those related to insurance coverage, reimbursement, and organizational policies. Similarly, a survey of Veterans Affairs providers identified low levels of knowledge regarding use of naloxone [19].

While these studies illustrate medical providers’ knowledge of *structural* harm reduction strategies such as MOUD and naloxone, little is known about how providers, including those working in social and administrative services, operationalize the principles of harm reduction into practice, i.e., *relational* harm reduction. Thus, in the current paper, we qualitatively explore HCW’s knowledge of and use of harm reduction via individual in-depth interviews, to operationalize how relational harm reduction is both characterized and employed in healthcare settings. As the current work is part of our larger study aimed at developing a harm reduction intervention for people with HIV (PWH) who use drugs, we focused on HCWs practicing in HIV care settings (citation redacted for peer review).

## Methods

### Sample and recruitment

Our study sample included three HIV clinics, one in Birmingham, Alabama (AL) and two in Pittsburgh, Pennsylvania (PA). We leveraged internal electronic messaging used by each site to disseminate information about the HCW interviews. IRB-approved recruitment messaging and a confidential link to the REDCap survey were included in the email messages, which were sent by site-level champions. HCWs were eligible if they (1) worked at one of these sites for at least one year and (2) provided “direct patient engagement” to PWH or PWUD at high risk for HIV acquisition. Eligible roles included front desk, clinical research coordinator, service coordinator, pharmacy, social worker, counselor/therapist, nurse, dietician, medical assistant, medical technician, advanced practice provider, physician assistant, and clinician. We included a wide range of HCWs rather than just licensed medical providers (e.g., clinician, nurse) to encompass the range of positions found at an HIV clinic, as modern HIV care is akin to a medical home model with coordinated medical and social services [20, 21]. We defined “drugs” as inclusive of illicit drugs and prescription drugs used in ways other than they were prescribed; we did not include alcohol or marijuana use, as these substances have been shown to carry less stigma than prescription drug misuse or use of drugs that are nationally criminalized [22, 23].

Individual interviews with *n* = 23 HCWs were conducted over a four-month period between November 2022 and March 2023. Interviews lasted between 30 and 60 min (average = 45 min). To maximize availability of the five study team members who led the qualitative interviews across Birmingham and Pittsburgh, interviews were conducted over HIPAA-compliant Zoom. Each of these study team members, including the PIs, three Co-Is, and a study coordinator, provided their availability on Microsoft Bookings. Interested HCWs could then sign up for an available time slot with a particular interviewer, thereby streamlining the recruitment process.

### Data collection

We used a semi-structured interview guide to explore health care workers’ attitudes towards and experiences with providing care to PWH who use drugs and their knowledge of and attitudes towards relational and structural harm reduction. We collected demographic information around participants’ racial and ethnic identity and gender identity, job title, and years of practice, including years specifically devoted to working with PWH, which we used to characterize the participant population in aggregate. Interviews were audio-recorded with participant permission and professionally transcribed verbatim. All identifying information was removed from study transcripts; each transcript was labeled with a numerical subject identification number and the information linking subject identification numbers with names was kept separate from the research records. All study activities were approved by the [name redacted] IRB.

### Analysis

Deidentified transcript data were uploaded into Dedoose [24] for analysis. We used a codebook thematic analysis to code the data, an approach to thematic analysis that fits within the positivist paradigm [25, 26]. One of the PIs (first author) read through each of the transcripts and familiarized themselves with the data. They reviewed field notes composed by study team members who conducted the interviews, which provided valuable critical reflection and interviewer feedback to inform analysis [27]. Then, this PI, in addition to four other members of the study team with expertise in qualitative analysis, independently coded three transcripts to identify broad themes. This team of five then met to discuss initial codes and resolve any discrepancies.

This list of initial codes was used to create a coding framework. We analyzed the data using both deductive coding from our interview guide (see Supplemental File) as well as inductive coding, achieving code saturation, which is typically attained from between 9 and 17 interviews with homogenous populations, well under our sample of *n* = 23 [28]. The first author and three other team members coded the remaining transcripts using this framework, meeting every two weeks and iteratively adding sub-codes and modifying the codebook using processes of adjudication. Five transcripts were double-coded (23%) and compared for consistency, following scholars’ recommendation to double-code between 10 and 25% of transcripts [29]. The final set of codes was combined into themes with the input of the full study team.

## Results

Twelve interviews were completed with health care workers in Birmingham, and 11 were completed with health care workers in Pittsburgh. Table 2 provides an overview of self-reported HCW characteristics.

**Table 2:** Characteristics of *n* = 23 health care workers who work at HIV clinics in Birmingham, AL and Pittsburgh, PA

Characteristic	Frequency (Percentage)
Race/Ethnicity	
White; Non-Hispanic	14 (63.7)
Black or African American	5 (22.7)
Hispanic/Latinx	1 (4.6)
Asian	1 (4.6)
More than one race	2 (9.1)
**Gender**	
Cisgender man	4 (18.2)
Cisgender woman	18 (81.8)
**Job Title**	
Pharmacist	1 (4.5)
Social Services Health Care Worker	4 (18.2)
Medical Services Health Care Worker	12 (54.5)
Administrative Services	5 (22.7)
**Number of Years Practicing Overall**	
1–5	6 (27.3)
6–10	4 (18.2)
11–15	2 (9.1)
16–20	5 (22.7)
20+	5 (22.7)
**Number of Years Working with PWH**	
1–5	8 (36.4)
6–10	7 (31.8)
11–15	2 (9.1)
16–20	2 (9.1)
20+	3 (13.6)

HCWs had a wide variety of ways that they both understood and practiced relational harm reduction in the HIV care setting with PWUD, with some not utilizing these harm reduction principles at all. We characterized this range of behaviors and attitudes, our primary analytical theme, *The Continuum of Relational Harm Reduction in Practice.* This primary theme had three subthemes: *Use of Relational Harm Reduction* characterized behaviors that corresponded to one or more of the six harm reduction principles. *Antithetical to Relational Harm Reduction* was characterized by HCWs who described an interaction that was at odds with the six principles of relational harm reduction. HCWs with *No Knowledge of Harm Reduction* were not familiar with harm reduction or its principles and had not integrated these principles into practice. An interrelated primary theme on *Limited Harm Reduction Training* was identified, in which HCWs discussed how they learned about harm reduction and source(s) of knowledge and highlighted the need for more training in this area. This theme helped to contextualize the primary theme by illustrating the extent to which the HCWs in our sample had been exposed to harm reduction education.

Illustrative quotes from both AL and PA are included for each subtheme to provide thick description and establish credibility of our findings [30]. Theme and subtheme prevalence is also provided; however, the importance of each theme or subtheme is not directly related to its prevalence. For each quote, we include both the location and HCW number; thus, for example, PA.1 would be the first HCW we interviewed who works at a Pittsburgh study site. We also provide participants’ specific job titles for each quote. Since there are a limited number of HCWs within each job category at our study sites, we do not include descriptives such as gender or race to protect participants’ confidentiality. [48]

### Theme 1: continuum of relational harm reduction in practice

#### Subtheme 1. Use of relational harm reduction

Nearly all HCWs (*n* = 19, 83%) described a patient interaction or expressed a sentiment that corresponded with one or more principles of relational harm reduction. These examples are further characterized and described below. While some of their harm reduction principles have natural overlap with each other (e.g., autonomy and individualism), we have identified principles that best reflect each illustrative quote.

##### Meeting patients where they are

Some HCWs described how they allowed patients to guide and lead their interactions rather than imposing a set agenda, as in the case of the following HCW who exemplified multiple principles of harm reduction (individualism, incrementalism, and pragmatism) in their commitment to assisting patients in their desired care plans:

I believe we do harm reduction every day…not showing or casting any kind of judgment on a patient, meeting them where they are and just listening, just trying to guide them through where you can and where they allow you to. (PA.7; Pharmacist)

This sentiment was expressed across HCW types in ways that reflected their respective job responsibilities. HCWs working in social services described how they helped patients access wraparound social services like housing or how they used motivational interviewing to “meet patients where they’re at.” This quote highlights the principles of autonomy and individualism.

I really just try to meet the patient where they’re at and with them making a decision on where they’re deciding to go next… And for me, I think, it’s always really just being here as a listening ear to the patient and making sure that they understand that I heard what they said and really try to figure out what can I do to help their experience be a little bit better and– even if it’s just for a moment. (AL 11; Therapist)

HCWs working in medical services focused on aspects of clinical care, as in the following quote that exemplifies both relational harm reduction (individualism and pragmatism) and structural harm reduction (MOUD induction):

I’m pretty good at kind of coaching patients through how to do it at home and, you know, they have good contact information with us should anything happen, should they need to talk to us. I kinda go over the different induction options with them, whether they wanna do traditional high dose or, um, low dose induction, and kind of just figure out what works for the patient. (PA.1; Clinician)

##### Knowing patients as humans

Most HCWs emphasized the importance of humanizing their patients (i.e., humanism) and getting to know them as people. This involved asking patients about their lives outside of their health condition(s) and creating a space where patients felt comfortable sharing personal details. An AL HCW discussed how they get to know their patients and their personal lives:

I feel like I know them on a more personal level. I know they’re aunties and their uncles and their dogs and their cats and, you know, everything about them because we talk all the time… I love that part [the personal connection]. (AL.3; Medical Social Worker)

A PA HCW shared how they start clinical visits by catching up:

You know, my first thing was, you know, “How have you been doing…We’ll get to your vitals and, you know, going over your medications, but how are you? Um, you know, how was your week?” You know, and it’s just, like, starting off like, “…I wanna make sure you’re okay.” (PA.2; Physician Assistant)

One HCW described this humanizing approach as “more important than what ailments our patients have.” HCWs also enjoyed having close relationships with patients. A PA HCW described their relationships with patient as a “privilege”: “When you see someone periodically every four to six months for years and you’re walking a journey with them, I consider a privilege to do. It’s very rewarding, and they– I think it’s mutual” (PA.10; Clinician). An AL HCW described their patients as more than “a number on a page”:

I really enjoy that patient interaction, is really getting to know people, versus see a number on a page. I really enjoy that aspect of even just finding a little snippet out about somebody. And since some of the people I see have been here in research for many years…when I see them sitting, they’ll be like, “Oh, hey, so-and-so” …some of my participants are still in other studies, so when they come in for a visit, they now know my face. We will speak. And they’re not just another person in the clinic. (AL.8; Clinical Research Coordinator)

##### Open communication

HCWs underscored the importance of creating an environment of mutual trust with patients where they felt comfortable disclosing drug use. This trust and open communication are hallmarks of the principle of pragmatism, which acknowledges that some patients will use drugs and the role of the HCW is to assist patients rather than offer judgments on their behaviors. This open communication also allows HCWs to give patients more tailored care that acknowledges the various circumstances in patients’ lives that can affect their health:

Um, you know, it should always be a no-judgment zone. You know, don’t pass judgments. You know, yeah, we want to know what’s going on with you, and it’s important for you to be honest because it only will allow us to, you know, individualize your care based off of what you’re dealing with. (PA.2; Physician Assistant)

HCWs also recognized the multiple and complex challenges that some patients faced and acted like “cheerleaders” whenever patients experienced setbacks, which relates to the principle of incrementalism:

*Interviewer*: What if they don’t make any progress at all or go backwards?

*HCW*: So I have some patients that are like that, and they’re like, “I almost didn’t come into my visit today because I was so upset that I have made no progress.” And I was– and then I just thank them for being honest, and that, you know, we can try again. …and then I talk about like, “Why did you not make progress? Like, was there a reason?” And they’re, you know, their mom died, and they got evicted. You know, and it’s like, “Okay. You had other things going on is reasonable that you were stressed out and that wasn’t the first thing on your list.” (AL.3; Medical Social Worker)

##### Always having the door open

HCWs discussed the importance of maintaining continuity of care with patients and not terminating anyone from care for continued drug use, which aligns with the principle of accountability without termination. An AL HCW shared how she was committed to “[getting] through this together” with patients:

One thing that I do like about our [Clinic] is they will, like, never terminate anyone. So a person– you know, like, they have all these other drugs in their subsystem, but they’re still able to come back. … I’ve had so many patients who’ve gone through treatment and relapsed again. I’m like, “It’s okay. You know, we’ll get through it together. You know, we can always try again. Like, you’re still here, and that’s the point. (AL.5; Clinician)

HCWs emphasized that their goal was to help patients, rather than “punish [them] for normal human behavior.” The only instances in which patients were terminated from care involved threats of violence or extreme verbal abuse:

*Interviewer*: Is there ever a point that you get to where you have to fire a patient?

*HCW*: If they threaten physical abuse in the clinic, or, you know, “I’m gonna kill.” You know, they’re really awful. Make racist comments to the front staff or, or just really do something totally egregious. If it’s moderately egregious, they get a warning letter, if it’s something awful, then they get a letter like, “You’re dismissed. Here are the other options for your care.” (PA.10; Clinician)

### Subtheme 2. Antithetical to relational harm reduction

Despite most participants integrating some aspect of relational harm reduction in their practice, over half (*n* = 14, 61%) also used language to describe PWH who use drugs that was stigmatizing or described an interaction that was antithetical to the six principles of relational harm reduction enumerated in Table 1—humanism, pragmatism, individualism, autonomy, incrementalism, and accountability without termination. These participants included HCWs from both AL and PA and encompassed a range of HCW types.

#### Substance use stigma

Substance use stigma was evident among nearly half of the HCWs (*n* = 10; 43%), although this was found to varying degrees. Some used stigmatizing terms like “addict,” but otherwise did not describe patients in a way that stigmatized their drug use. Others characterized PWUD in a broadly negative sense that went beyond terminology, such as in the following quote from a PA HCW: … “Your mother’s an addict too and you know, half your family are addicts, so it’s, it’s just–I think that’s probably the biggest thing is the social chaos. It, uh, kind of– kind of flies around people who have problems with addiction.” (PA.2, Clinician). For other HCWs, stigma was evident in the way they framed abstinence as “bettering yourself,” becoming a “productive member of society,” etc., without consideration of around whether abstinence aligned with the patient’s own goals or what these goals looked like in general. As an example, one HCW described their work with PWUD and a need for these to patients to “change”:

A lot of them, from whom I’ve spoken with, they don’t like change. They just kinda want everything to remain the same. And unfortunately, in life, you know, you’re gonna have change… unfortunately, sometimes, they fall by the wayside. And they need that force to kind of pick them back up and to help them guide them along a little bit to come back on the narrow path. (PA.6; Medical Assistant and Health Coach)

Other HCWs made negative generalizations about PWUD’s ability to take their HIV medication as directed, as well as their health in general:

The substance use interferes with their ability to take their medication as directed, and it also interferes with their ability to eat in a way that’s generally healthy…it seems to be that people with active substance use will have higher viral loads and lower CD4 counts which is indicative of not taking the medication every day. (AL.10, Registered Dietician)

Similarly, a nurse from AL described PWH who use drugs as unable to stay adherent to HIV medication:

So they will prioritize their next fix over trying to stay as healthy as possible or not transmitting HIV to someone else. And they’ll actually trade sex for that next fix, and they’re not biologically suppressed, so.” (AL.1, Nurse)

In these examples, substance use stigma is evident in the ways that HCWs describe PWUD and minimize their individualism and autonomy by making sweeping (and negative) generalizations.

#### Characterizing abstinence as the end goal for all PWUD

A key tenet of harm reduction is an understanding that patients’ goals might not reflect that of HCWs’, and that abstinence may not be the goal for all PWUD. However, some HCWs (*n* = 7; 30%), primarily in AL, described harm reduction as a kind of stepping-stone on the way to abstinence, implying that abstinence is the only acceptable outcome for patients:

I mean, harm reduction is a part of soberness. I feel like it’s the first step to getting people to a point where they’re willing to contemplate changing, and making them more aware of what they’re actually doing…because in order to practice harm reduction, you have to be aware that you need to. And if you’re aware that you need to, then you’re aware of what you’re doing, and you’re aware that it could cause problems. And if you get to that point, then you might be willing to talk about further initiatives for change. (AL.3; Medical Social Worker)

This emphasis on abstinence was seen in the way some HCWs couched patients as “taking advantage” of harm reduction, despite it being a medical resource:

The only time I have a problem with [harm reduction] is when I think people are, like, really taking advantage of it, that they’re just manipulating the system because they know we’re harm reduction. I don’t see that in the majority of our patients, but, you know, I definitely have seen it a few times, and it makes me, like, upset because I’m like, “We’re doing everything we can do for you, and you’re still, like, either not listening or not, like, doing what you, you need to be doing.” (PA.8; Nurse)

Similarly, several AL HCWs felt there should be a limit to harm reduction. One HCW was uncertain about the role of Suboxone and whether it should be taken indefinitely:

I do believe that the end goal [of harm reduction] could at least could be abstinence because I feel like, you’re addicted to opioids, and you want to get off that to kind of switch Suboxone, and to me, that’s like, “Okay, you’re taking the steps towards, um, you know, being abstinent and–” but it like kind of stops there. It’s like, you just take Suboxone for the rest of your life…I don’t know. And I feel like the [clinic] doesn’t really stress the importance of abstinence. They are really just strictly harm reduction, which is fine, but I think there also needs to be like a second, um, part of it of abstinence. (AL.12; Social Worker)

Another AL HCW shared this sentiment, feeling like there should be a limit to harm reduction: “There’s a little bit of a fine line with harm reduction. And I think some of our patients may feel like we need to just give them whatever they want.” (AL.1, Nurse).

### Subtheme 3. No knowledge of harm reduction

Though reflecting a minority of participants, several HCWs (*n* = 5, 22%; all from Birmingham, AL) were unfamiliar with the term “harm reduction.” However, after being given a standardized definition from the interviewer, some HCWs were able to relate it back to their work. For example, a dietician shared how they could use harm reduction in their practice:

I kind of use harm reduction in nutrition because I hope to help people balance, right? So people who don’t want to give up sodas, for example, maybe we can think of something else they could do to, to, you know, reduce that harm, like, walking more, whatever. (AL.10; Registered Dietician)

Similarly, an administrative HCW working with dental patients identified how harm reduction could relate to dental care for PWUD, noting that patients could decrease drug use to protect their dental health: “It’s not like, you know, like, “Oh, you gotta stop [using drugs] tomorrow,” no. But, like, you know, “Step by step, you know, start, like, you know, thinking about, you know, your, your, your smile.” (AL.6, Medical Technician).

### Theme 2: limited harm reduction training

A minority of HCWs in our study had been exposed to harm reduction education (*n* = 7; 30%). None of the HCWs had received any formal training in harm reduction while in school, while just one clinician from AL had received training through a fellowship. Most HCWs had little experience working with PWUD prior to their current roles and were not familiar with harm reduction outside of structural services, despite most HCWs describing interactions with patients that aligned with one or more principles of relational harm reduction. Overall, HCWs in AL had less exposure to harm reduction than HCWs in PA. In fact, only three HCWs (two clinicians, one nurse) in AL had ever received any harm reduction education. This education came from a fellowship, webinars, conferences, and even social media. As AL.5 noted, X (formerly known as Twitter) was their primary source of information post-fellowship:

Yeah, a lot of it has been me, um, just reading on my own and following– I mean, frankly, a lot of it I get through Twitter. A lot of the people I follow on Twitter are harm reductionists, addiction medicine docs, infectious disease docs who do addiction medicine work. That’s where I’ve learned a lot about clinical practice policy

AL HCWs also discussed the importance of exposing more medical students to harm reduction to “destigmatize” working with PWUD:

And you’re right, it’s not really trained. At least I wasn’t trained, in the way that I practice now, when I was in residency or fellowship. There was no substance use clinic that I could shadow at or kind of rotate through when I was in training. And I wish there was. And we certainly have the fellows come and experience [Clinic] now when we have residents and PA students. We try to get as many learners as possible for this reason, that we want to open their eyes…to this whole kind of population in need that is, and it’s really satisfying work. (AL.5; Clinician)

HCWs in PA (n = 5) described receiving structural harm reduction education at multiple levels of influence, including the healthcare network (healthcare network-wide trainings), organization (learning on-the-job), and patient levels (learning about harm reduction from the patients themselves). These HCWs came from a variety of backgrounds, including a counselor, nurse, benefit services coordinator, clinical research coordinator, and clinician. A clinician described how their work with patients made them a better harm reductionist and demonstrated use of both structural and relational harm reduction in their practice:

Just constantly be getting feedback from the people you’re helping and what they think about something as simple as, like, really, “I hate this brand of syringes. Like, they keep breaking. They don’t [plunge], and, like, that kinda feedback. Really trying to make it comfortable for everybody and just learn as you go. (PA.9; Clinician)

This same clinician further discussed that they, like their colleagues, had “picked [harm reduction] up along the way:

…Like a lot of people in addiction medicine, kind of picked it up experientially, just like a lot of people back in the day with HIV when it wasn’t part of any kind of training. It was just, you know, something people did… So I just kind of picked it up along the way. (PA.9; clinician)

One HCW characterized harm reduction as being innate rather than something that needed to be taught:

I guess [harm reduction]’s not something that can be taught…it’s all about, you know, caring about the next person, no matter what they’re dealing with, wanting to see everybody succeed, even, like, professionally. (PA.2; Physician Assistant)

## Discussion

These qualitative findings reveal the extent to which relational harm reduction exists as a continuum in HIV care settings. Harm reduction can even occur along a continuum for a single HCW, as evidenced by individual HCWs in our study who used stigmatizing terms such as “addict” or “drug abuse,” for example, but who also described patient interactions that reflected principles of relational harm reduction. These contradictions highlight the complexity of providing harm reduction care and the fact that no HCW is “perfect.” Interestingly, we did not identify any place- or HCW role-based trends in HCWs’ use of relational harm reduction and integration of the six principles in practice, suggesting that this continuum may exist despite variation in legality of harm reduction services (e.g., syringe services not legal in AL but are legal in Pittsburgh, PA).

Our study also identified the need for more relational harm reduction training. Few HCWs had received any formal education in either relational or structural harm reduction. AL HCWs were primarily self-taught and discussed the paucity of training, which is likely a result of having fewer resources for PWUD, including a lack of legalized sterile syringe programs (SSPs) [2]. However, even HCWs in Pittsburgh, where SSPs operate legally [31], primarily learned on-the-job rather than via a formal training program. Interestingly, while PA HCWs spoke about learning of the ethos of harm reduction from their clients, AL HCWs did not identify clients as a source of harm reduction knowledge. Another difference between the two groups of HCWs was that only PA HCWs discussed harm reduction as an innate method of caring for PWUD. While it is difficult to speculate on the reasons for these setting-based differences that are not a direct reflection of policy context, it may be that policy-level differences had a downstream effect, such that AL HCWs felt less empowered or confident as harm reductionists given more limited resources. However, some HCWs who stated that they were not familiar with or had not been trained in harm reduction were able to provide examples of relational harm reduction in practice, suggesting that some HCWs may be practicing harm reduction without recognizing it as such. Given that harm reduction is typically described as a structural approach, a broader definition of harm reduction that is not dependent on policy-dependent resources is needed.

HCWs emphasized the importance of getting to know their patients as human beings beyond their health diagnoses (humanism). This led to enhanced patient comfort and a sense of fulfillment for HCWs. A personalized relationship has been identified as one of the strongest independent predictors of adherence to antiretroviral therapy for PWH [32]. The importance of the patient-provider relationship and the significance of providers earning patients’ trust in harm reduction and substance use treatment settings has also been recognized in extant literature [33–35]. Yet, less is known about the extent to which humanism impacts substance use-related health outcomes. Additional research is needed to explore this potential relationship.

Harm reduction does not preclude abstinence and may be a treatment goal for some PWUD. The emphasis on abstinence for all PWUD among some HCWs in our study, and even the suggestion that MOUD should be a time-limited healthcare service despite the wide evidence base to the contrary [36], is reflective of a larger cultural emphasis on sobriety and the widespread criminalization of drug use. Scholars have noted that moralism pervades anti-harm reductionist views, and that, despite an economic and medical evidence base supporting harm reduction [37], a belief that drug use is “immoral” diminishes support for harm reduction policies and programs [38]. Favoring abstinence may also decrease support for harm reduction programs [39]. Yet, as our study demonstrates, support for harm reduction and attitudes towards abstinence may not always be linearly related, and harm reduction often exists on a continuum. Even organizations that officially incorporate harm reduction may still favor abstinence and stigmatize people who are actively using drugs. For example, a qualitative study of staff and residents at a housing first program described how abstinence was characterized as “improving one’s life” and emphasized the importance of “getting clean” [40]. Participants also spoke about the disconnect between policy and practice, in which abstinence was not required for program entry but substance use onsite was not tolerated and could lead to dismissal [40]. Similarly, while all HIV clinics included in our study directly provided or referred patients to harm reduction services, abstinence was prioritized among some of the HCWs and clearly pervaded their interactions with patients.

### Limitations

These qualitative findings reflect the perspective of HIV HCWs in Birmingham, AL and Pittsburgh, PA, and may not reflect the attitudes of HCWs who work outside of HIV clinics or elsewhere in the United States. Recruitment language shared with HCWs stated that “the aim of this study is to understand the ways that harm reduction care and stigma experienced in healthcare settings affect clinical outcomes for people living with HIV who use drugs.” As a result, HCWs who elected to participate in the interviews may have been more knowledgeable about harm reduction than those who did not. These perspectives may therefore not be representative of those of HCWs less familiar with harm reduction work. However, results demonstrated a wide range of harm reduction approaches and variable familiarity with the concept, suggesting that our sample was fairly heterogeneous.

## Conclusion

Our study is the first, to our knowledge, that explores how HIV HCWs utilize relational harm reduction in HIV primary care settings. With this work we seek to amplify voices of those with lived experiences by explicating ways that the long-standing principles of harm reduction, which were developed by PWUD for PWUD, can be translated to and practiced in healthcare settings regardless of policy contexts that may enable or inhibit structural harm reductions strategies such as syringe services or MOUD. Our findings suggest that relational harm reduction in our study settings is practiced along a continuum. Some HCWs were experienced in integrating relational harm reduction into their interactions with patients, while several HCWs were entirely unaware of harm reduction. Interestingly, we also found that even strong harm reductionists shared sentiments or used language in opposition to harm reduction principles, suggesting that even experienced HCWs could benefit from additional training. Given the health benefits associated with harm reduction care, additional research is needed to identify ways to strengthen harm reduction approaches in HIV settings.

### Electronic supplementary material

Below is the link to the electronic supplementary material.

Supplementary Material 1

## Data Availability

No datasets were generated or analysed during the current study.

## Abbreviations

AL: Alabama
ART: Antiretroviral Therapy
HCW: Health Care Worker
MOUD: Medication for Opioid Use Disorder
PA: Pennsylvania
PWH: People With HIV
PWUD: People Who Use Drugs
SSP: Syringe Service Program
SUD: Substance Use Disorder
