# Supplementary material for: A qualitative exploration of health care workers’ approaches to relational harm reduction in HIV primary care settings
Source: Harm Reduct J. 2024 May 17;21:97. doi: 10.1186/s12954-024-01021-x (PMC11100089; doi:10.1186/s12954-024-01021-x)
Supplement: Supplementary file 1 — Supplementary Material 1 [file 12954_2024_1021_MOESM1_ESM.docx]

**Health Care Worker Qualitative Interview Guide**

1. Describe your role at [Clinic]. Also tell me a bit about the patient population you see.
   - How is your time spent at the clinic?
     1. Patient care
     2. Teaching
     3. Other?

- How are your patients at [Clinic] the same or different from other places you’ve worked?
  - What’s unique about working with PWH who use drugs?
  - Probe for research positions as applicable
    1. How is this different from other clinical care, what does patient interaction look like?
    2. In a typical week, how often do you see patients? How much time do you spend with them at visits?
    3. Does your site empanel patients?

1. Relational aspects of care
   - 1. What things do you typically do during visits with patients?
     2. Tell me about a really good interaction with a patient? A really bad one?
     3. How much do you know about your patients’ lives?
        1. How often do you talk with your patients about things outside of clinical care?
        2. Beyond clinical care, how do you learn about your patients’ lives?
        3. Why did you become a(n)….
        4. In your mind, what is the ideal relationship between providers/patients?
2. When you talk with people outside of [Clinic] about the work that you do, how do you describe it?
3. I’d like to know more about your experience working with people with HIV who use drugs.
   - What kinds of things have helped you do this work? [e.g., clinical training, continuing education, coursework, self-taught]
   - How comfortable or uncomfortable are you working with this population?
     1. Follow-up: Has this changed over time? Did you do anything in particular that helped you feel more comfortable?
   - In some of the survey responses we got from different sites, we learned that sometimes providers find it challenging to work with people who use drugs. Do you agree? What do you think drives that?
   - What about benefits of working with this population? What are some of things you like about working with this community?
4. How do you make use of the substance use resources in your Clinic? Community?

- Describe how referral works to your community collaborators.
- Tell me about access to sterile syringes. (Probe to assess if they are aware if SSPs are legal or not.)

1. What happens when patients who inject drugs ask you about how to use safer?
   - Probe (If they go right to referrals): What are those conversations like?
2. In your experience working at [Clinic], have you noticed any differences in the way White and Black PWH who use drugs are treated?
   - Without using names, describe any instances of racial discrimination you have witnessed or heard about.
   - Again without using names, describe any provider or clinic staff racial biases you are aware of.
3. What are the service gaps for PWH who use drugs in your (a) clinic or (b) community?
   - What do you think is the number one barrier to care for PWH who use drugs?
   - What other barriers to care do PWH who use drugs face?
   - How can we improve health outcomes for PWH who use drugs?
4. I’d like to transition a bit and talk about harm reduction specifically. Tell me about your experience with harm reduction, or just what you know about it. [If they do not know what harm reduction is, can use this definition; *Harm reduction refers to interventions aimed at reducing the negative effects of health behaviors, without necessarily eliminating them entirely.)*
   - What kinds of training specific to harm reduction have you had?
   - What are your thoughts about this approach to care?
   - [If only structural harm reduction is mentioned]: Harm reduction also has to do with the way providers interact with their patients. What are your thoughts on that?
5. Is there anything about working with PWH who use drugs that I didn’t ask about but is important for me to know?
   - Is there anyone else you think we should talk with?
